# A Whole-Chromosome Analysis of Meiotic Recombination in *Drosophila melanogaster*

**DOI:** 10.1534/g3.111.001396

**Published:** 2012-02-01

**Authors:** Danny E. Miller, Satomi Takeo, Kavyasree Nandanan, Ariel Paulson, Madelaine M. Gogol, Aaron C. Noll, Anoja G. Perera, Kendra N. Walton, William D. Gilliland, Hua Li, Karen K. Staehling, Justin P. Blumenstiel, R. Scott Hawley

**Affiliations:** * Stowers Institute for Medical Research, Kansas City, MO 64110; † Department of Biology, Depaul University, Chicago, IL 60604; ‡ Department of Ecology and Evolutionary Biology, University of Kansas, Lawrence, KS 66045, and; § Department of Physiology, Kansas University Medical Center, Kansas City, KS 66160

**Keywords:** crossing over, gene conversion, double-strand break, genome sequencing, meiosis

## Abstract

Although traditional genetic assays have characterized the pattern of crossing over across the genome in *Drosophila melanogaster*, these assays could not precisely define the location of crossovers. Even less is known about the frequency and distribution of noncrossover gene conversion events. To assess the specific number and positions of both meiotic gene conversion and crossover events, we sequenced the genomes of male progeny from females heterozygous for 93,538 *X* chromosomal single-nucleotide and InDel polymorphisms. From the analysis of the 30 F1 hemizygous X chromosomes, we detected 15 crossover and 5 noncrossover gene conversion events. Taking into account the nonuniform distribution of polymorphism along the chromosome arm, we estimate that most oocytes experience 1 crossover event and 1.6 gene conversion events per *X* chromosome pair per meiosis. An extrapolation to the entire genome would predict approximately 5 crossover events and 8.6 conversion events per meiosis. Mean gene conversion tract lengths were estimated to be 476 base pairs, yielding a per nucleotide conversion rate of 0.86 × 10^−5^ per meiosis. Both of these values are consistent with estimates of conversion frequency and tract length obtained from studies of *rosy*, the only gene for which gene conversion has been studied extensively in *Drosophila*. Motif-enrichment analysis revealed a GTGGAAA motif that was enriched near crossovers but not near gene conversions. The low-complexity and frequent occurrence of this motif may in part explain why, in contrast to mammalian systems, no meiotic crossover hotspots have been found in *Drosophila*.

*This article is dedicated to the memory of Professor Arthur Chovnick, whose studies of the rosy locus form the foundation of our understanding of gene conversion in Drosophila*.

A hallmark of most meiotic processes is the induction of a substantial number of programmed double-strand breaks (DSBs) that result in meiotic recombination between homologs. A portion of these DSBs are repaired as crossover (CO) events that result in both gene conversion (GC) at the site of the DSB and reciprocal exchange between flanking markers. Alternatively, DSBs may also repair without crossing over, in which case GC may still occur, but the exchange of flanking markers does not (Berchowitz and Copenhaver 2010; Bishop and Zickler 2004; Youds and Boulton 2011). Both forms of recombination are key determinants of diverse biological phenomena that include the dynamics of chromosome segregation, patterns of linkage disequilibrium (LD), and the evolutionary fate of beneficial alleles. However, very little is known about the molecular mechanisms that determine the landscape of meiotic recombination across species. In fact, very little is known about how meiotic recombination is distributed across the genome at a fine scale. In this study, we seek to provide insight into these questions from a *Drosophila* perspective by using the tools of whole-genome sequencing (WGS).

In *Drosophila* females both the number of DSBs induced per meiosis (~21) (Jang *et al.* 2003; Klovstad *et al.* 2008; Mehrotra and McKim 2006) and the number of CO events per genome (~5−6) (Carpenter 1975) are well established. Unfortunately, although GC has been thoroughly studied at the *rosy* locus (Blanton *et al.* 2005; Chovnick *et al.* 1971; Curtis and Bender 1991; Curtis *et al.* 1989; Hilliker *et al.* 1994; Radford *et al.* 2007b; Schweitzer 1935), only limited data are available for either conversion or intragenic recombination at other loci (Finnerty 1976; Green and Green 1949; Smith *et al.* 1970), raising the possibility that genome-wide estimates of conversion rates may be inaccurate if the frequency of conversion at *rosy* is substantially different from the true genome average. Of equal concern is the paucity of data regarding the precise localization of CO events within the genome. Finally, although the classical analysis of crossing over using multiply marked chromosomes does not indicate the presence of significant hotspots for CO events (Hey and Kliman 2002; Lindsley and Sandler 1977; Schweitzer 1935), the resolution of such studies may be insufficient to identify such sites.

Beyond the classic approach of using visible markers, two basic approaches can be taken to define the landscape of meiotic recombination with high resolution. The first approach leverages population genetic data and patterns of LD to infer ancestral recombination events. This approach has proven extremely powerful, especially in humans (Hinch *et al.* 2011; McVean *et al.* 2004; Myers *et al.* 2005, 2008). One limitation of this method, however, is that it might be confounded by natural selection because selective sweeps can remove the ancestral signature of recombination faster than drift (O’Reilly *et al.* 2008). In addition, such methods are unable to easily distinguish recombination arising from single COs (SCOs), double COs (DCOs), or non-CO GC events (hereon designated GC events). The ability to make this distinction is essential for understanding the mechanisms that determine the landscape of recombination.

In contrast to the population genetic approach, a second approach to analyzing the landscape of recombination at high-resolution has been a pedigree-based approach. In the most basic form, this approach depends on genotyping parents and offspring, thus defining the landscape of recombination in a single meiosis. This approach has also proven very powerful in humans (Kong *et al.* 2002, 2010) and in yeast (Mancera *et al.* 2008). Similar methods have been used in *Drosophila* via two distinct approaches. First, Noor and colleagues, have used large numbers of single-nucleotide polymorphism (SNP) markers to detect recombination events and thus construct fine-scale recombination maps in *Drosophila pseudoobscura* and *Drosophila persimilis* (Kulathinal *et al.* 2008; Stevison and Noor 2010). Second, Singh *et al.* 2009 have used an approach that identified recombination events in *Drosophila melanogaster* within the *white-echinus* interval and then determined their precise location within this interval. However, in the first case, resolution was not sufficient to address important questions such as whether CO or GC events occur more frequently than expected in intergenic regions, or in regions marked by specific sequence elements or motifs. Similarly in the Singh *et al.* (2009) study, it was not clear how the identified fine-scale heterogeneity observed in the interval studied would be distributed over the entire genome.

Addressing such questions requires the ability to detect all CO and GC events along a large fraction of the genome, preferably over at least one whole chromosome arm, in the product of a single meiosis. Such an experiment is also best performed using sets of parents, all of which are heterozygous at the same sites, which is to say sibling females created by the mating of recently isogenized stocks. Provided that the two parental stocks differ by a sufficient number of DNA polymorphisms, sequencing of individual progeny should be able to precisely identify CO and GC events by WGS, with a resolution limited only by the number and distribution of those polymorphisms (Qi *et al.* 2009). Theoretically, more precise recombination estimates may be obtained from an analysis of progeny that are descendants of several generations because this allows more recombination events to be detected. However, in our case, single meiotic events were preferred because of our desire of being able to distinguish SCO, DCO, and GC events. If COs have a tendency to cluster as the result of recombination rate heterogeneity, it is extremely difficult to distinguish among these three forms of recombination after multiple rounds of meiosis.

Despite the great progress in genome sequencing (Metzker 2010; Nielsen *et al.* 2011), several challenges remain in taking a whole-genome approach to analyzing the distribution of recombination events from a single meiosis. First, assembling and scoring heterozygous reads is difficult and requires much deeper sequencing than required if simply scoring homozygous SNPs (Langley *et al.* 2011). Second, a sufficient amount of DNA may not be available from single F1 progeny to avoid whole-genome amplification (WGA), which may be susceptible to artifacts (Pugh *et al.* 2008). If follow-up verification of recombination events is desired, it may be preferable to use a sample of DNA that has not been subjected to WGA.

We have circumvented these problems by focusing our attention on *X* chromosomal recombination. In *Drosophila*, the *X* chromosome carries nearly 20% of the euchromatic genome sequence and is hemizygous in males. Thus, by sequencing male F1 progeny, the difficulty of dealing with heterozygous SNPs can be avoided by focusing strictly on recombination on the *X*. Restricting the analysis to the *X* chromosome also allows putative sequence changes to be confirmed without WGA because the *X* chromosome can be preserved and “cloned” by crossing the single male progeny to attached-*X C(1)DX/Y* females, hereon designated *C(1)DX*, and creating stocks (supporting information, Figure S1). An alternative approach would be to use balancer chromosomes and an appropriate crossing scheme that would allow one to feasibly purify recombinant autosomes in an isogenized state. However, because we have previously shown that there is a detectable amount of GC between balancer and normal sequence chromosomes (Blumenstiel *et al.* 2009), we elected to avoid this approach.

The two isogenic stocks we used as parental lines, Canton-S and *w^1118^*, differ by 93,538 SNPs and InDel polymorphisms on the 22.4-Mb *X* chromosome, allowing us a to detect virtually all CO events and, on the basis of statistical predictions of the SNP distribution, approximately 40% of all GC events assuming an average conversion tract length of 476 bp. Putative CO or GC events identified by WGS can be confirmed by using standard methods to resequence the relevant intervals from the preserved *X* chromosome in each of the stocks.

Using this approach, we precisely localized 15 CO events and 5 GC events on the *X* chromosome arising from a total of 30 meiotic events. This allowed us to position CO events within relatively small regions, on average, 923 bp. We were also able to determine the maximum and minimum tract lengths of the five GC events, with the largest possible conversion tract comprising 3436 bp and the narrowest possible tract comprising 87 bp. Our data suggest that the previous genetic studies of GC at the *rosy* locus provide an excellent estimate of the incidence of GC throughout the genome, as well as the average tract lengths of those events (Blanton *et al.* 2005; Curtis and Bender 1991; Curtis *et al.* 1989; Hilliker *et al.* 1994; Radford *et al.* 2007a, 2007b). Interestingly, we have found that CO spans are enriched for a simple GTGGAAA motif. The low complexity of this motif suggests a possible reason for why no strong recombination hotspots have been found in *Drosophila* since this motif is broadly distributed along the *X*.

As read lengths increase and the cost of WGS decreases, we expect that this approach for the analysis of recombination and conversion will become a standard method for the analysis of recombination-defective mutants in *Drosophila*.

## Materials and Methods

### Fly genetics

*w^1118^* and Canton-S parental lines were isogenized for the *X*, second, and third chromosome using balancer stocks. *FM7* was used to balance the *X* chromosome and a *w; Sp/CyO; Pr Dr/TM3* stock was used for crosses in which it was necessary to balance the second and third chromosomes. *FM7* is a balancer chromosome that carries the markers *y*, *w^1^*, and *B* and completely suppresses crossing over with a normal *X* chromosome. *w; Sp/CyO; Pr Dr/TM3* is a double balancer stock where the *CyO* chromosome carriers the marker *Cy* and the *TM3* chromosome carries the marker Sb. The genotype of the fourth chromosome was not considered. *w^1118^* and Canton-S stocks isogenized for the *X*, second, and third chromosomes were then sequenced, and male *w^1118^* were crossed to Canton-S females to generate females for analysis of female recombination by sequencing their male progeny. The full cross is shown in Figure S1. Before sequencing was initiated, recombinant *X* stocks were established by crossing single male progeny to four *C(1)DX* females for 5 days before the male was removed and stored at −80°. The females were removed and discarded on the eighth day. Male progeny were collected from each stock as needed for sequencing the recombinant *X* chromosome within the stock.

### Genome sequencing, SNP genotyping, and mapping recombination events

DNA was prepared from 10 adult males using standard procedures (Blumenstiel *et al.* 2009). A size of 300 bp of genomic DNA was selected after shearing to 200- to 800-bp fragments using sonication and DNA fragment libraries were made using the Paired End DNA Sample Prep Kit (cat. no. PE-102-1001) from Illumina following the manufacturer’s directions. The libraries were then sequenced using 40-bp paired-end reads on an Illumina Genome Analyzer IIx. Two lanes were run for each of the parental lines, and one lane was run for each of the 30 progeny lines. One lane of each flowcell was used for a Phi-X control. Images from the Illumina Genome Analyzer IIx were processed using the Illumina Analysis Pipeline version 1.6.0 (Casava) to generate FASTQ sequence files. Reads that passed through the Gerald chastity filter were aligned to the *D. melanogaster* genome (Release 5.22) using MAQ 0.7.1 (Li *et al.* 2008), allowing two mismatches in the first 24 bps and a maximum mismatch quality sum of 60. A consensus was then generated using Samtools pileup (Li *et al.* 2009) for each strain. Consensus sequences for the Canton-S and *w^1118^* isogenized strains were compared to identify differences. Local Smith-Waterman alignments were performed using GATK (Depristo *et al.* 2011; McKenna *et al.* 2010) in regions flanking the initially identified SNP to improve the alignment quality. To call a difference, a minimum MAQ consensus quality score of 30 and read depth of 4 were required at each chromosomal position for both strains. Recombination events were identified using custom PERL or R scripts that locate sites of putative CO over or GC. Most COs and all GCs were verified using Sanger sequencing on PCR products derived from individual males from both parental lines (Canton-S and *w^1118^*) and the recombinant *X* stocks.

### Analysis of clustered recombination events

The likelihood of seeing two CO events only 25 kb apart was estimated by generating 10,000 sets of 15 random coordinates between 1 and 1.4 × 10^7^ (14 Mb was used instead of 22 Mb to account for CO events occurring primarily in the distal two-thirds of the *X* chromosome), and the smallest distance between adjacent coordinates in each set was then calculated. This showed that the average smallest distance between any pair of 15 coordinates was approximately 65 kb and that the smallest distance was ≤25kb 30.9% of the time. Nonetheless, the possibility of increased recombination rate in several positions was further tested using polymerase chain reaction (PCR)-based restriction enzyme analysis. A total of 96 male progeny were created by crossing isogenized male *w^1118^* and female Canton-S parents as described, crossed to *C(1)DX* females, and homogenate for PCR was prepared from the resulting male progeny as described. Three pairs of SNPs were identified that flanked the three intervals that showed more than one CO (8C1: 8,762,204–8,915,350. 11A3: 11,776,391–12,129,120. 18C3-18D1: 19,238,119–19,498,335: Release 5.22; Tweedie *et al.* 2009) and resulted in restriction sites in only one of the two parental lines. Six pairs of PCR primers were designed to create approximately 1-kb products centered on each SNP. Each of the 96 males was then scored for the presence of a recombination event in the relevant interval.

### Statistical analysis of recombination

CO and non-CO GC (designated as GC) frequencies were analyzed separately for several reasons. First, they occur by mutually exclusive pathways (Berchowitz and Copenhaver 2010; Bishop and Zickler 2004; Youds and Boulton 2011). Second, in the case of CO, all recombination events are visible because of the exchange of flanking markers. In contrast, not all GCs are visible because they can occur entirely between two flanking SNP markers. Thus, a means of correcting the estimate applies solely to GCs. Finally, no CO in this experiment was associated with a discontinuous pattern of exchange, indicating that GC tracts to the left and right of a DSB are most often contiguous. Without sequences from the products of reciprocal exchange, there is no power to estimate GC tract lengths that arise during crossing over. Thus, tract length estimates were obtained using GCs only.

For GCs, we used a maximum likelihood approach to jointly estimate frequency of GC DSBs per nucleotide per meiosis and the length of the GC tracts. In the case of GC DSBs, we modeled their position to be Poisson distributed between nucleotides with parameter λ. We modeled GC tracts based on a geometric process with parameter *p* (Blanton *et al.* 2005; Hilliker *et al.* 1994) that occurs independently to both the left and right of the DSB. In this case, *p* represents the probability that a GC tract ceases to extend after *L* nucleotide extensions, each with probability (1 *− p*). Thus, the probability of either a leftward or rightward GC tract being exactly length *L* is given by 1)

$$P(GC=L)={\left(1-p\right)}^{L}p$$

And the probability of a GC being in a specified range is the difference between the cumulative density function for the maximum value of this range subtracted by the cumulative density function for the minimum value of this range. This models the process of 5′ resection that occurs at the sites of DSBs and gives a mean leftward or rightward GC tract as (1 − *p*)*/p*. In the case of neighboring SNPs that were not converted, we modeled the likelihood as equal to the probability of no GC occurring in the span between the unconverted SNPs plus the probability of exactly one GC within the span, but with GC tract lengths short enough so as to not have converted the flanking SNPs. Thus, the probability of a given span with no converted flanking SNPs is 2)

$$P_{NoGCSpan}=\frac{{\left(i\lambda \right)}^{0}}{0!}e^{-i\lambda }+\frac{{\left(i\lambda \right)}^{1}}{1!}e^{-i\lambda }\sum_{k=1}^{i}\frac{P_{k}(GCTractMaxLeft)P_{k}(GCTractMaxRight)}{i}$$

where *i* is the distance between flanking SNPs (*i.e.* the number of possible DSB locations) for the single DSB and *k* indicates the ordinates for positions that a DSB may occur within the span.

In the case of SNPs that experience GC, the likelihood was modeled such that a single DSB could have occurred anywhere between the two outermost unconverted SNPs. Within this span, there may be either one or multiple converted SNPs. Depending on the possible location of the DSB, to the left and right the probability of observing a particular GC conformation was determined based on the maximum possible GC (bounded by unconverted SNPs) and the minimum possible GC (bounded by converted SNPs). Assuming the probability of two DSBs within the span is negligible, these probabilities were summed over all positions within the span 3)

$$P_{GCSpan}=\frac{{\left(i\lambda \right)}^{1}}{1!}e^{-i\lambda }\sum_{k=1}^{i}\frac{\left[P_{k}(GCLeft)][P_{k}(GCRight)\right]}{i}$$

where:

$$\begin{array}{l} P_{k}(GCLeft)=P_{k}(GCTractMaxLeft)-P_{k}(GCTractMinLeft)\\ P_{k}(GCRight)=P_{k}(GCTractMaxRight)-P_{k}(GCTractMinRight) \end{array}$$

Given this formulation, there is a unique probability for each span between two unconverted SNPs of distance *i* and a unique probability for each observed GC. The full likelihood of the data are this given by 4)

$$L(Data)=\prod_{a=1}^{b}{\left(P_{a.NoGCSpan}\right)}^{N_{a}}\prod_{c=1}^{d}P_{c.GCSpan}$$

Where *N_a_* is the cumulative number of spans of length *a* that were witness to no GCs across all 30 meioses and *b* is the upper limit for span lengths used in the analysis. In this case, we set *b* to 10,000, meaning we did not include spans between SNPs greater than 10,000. Such spans yield very little information on the GC process and were thus excluded. In the second part of the likelihood function, *c* is the index for the GC tracts and in this case *d* is five. Taking the natural log of this gives 5)

$$\ln L(Data)=\sum_{a=1}^{b}N_{a}\ln(P_{a.NoGCSpan})+\sum_{c=1}^{d}\ln(P_{c.GCSpan})$$

Using this function, we jointly determined the values of *p* and λ that maximized the likelihood of the data. All analysis was performed in *Mathematica* (Wolfram Research). Then, 95% confidence intervals (95% CIs) for estimates were determined on the basis of determining parameter values that gave likelihood scores ± two ln-likelihood units while keeping the second parameter fixed for the maximum likelihood estimate. Tests of significance against previous estimates were performed using the likelihood ratio test and the χ^2^ distribution with degrees of freedom determined based on the number of additional parameters allowed to freely vary.

### CO motifs

We used MEME (Bailey and Elkan 1994, 1995a; Bailey *et al.* 2006) to identify candidate motifs in the 14 CO spans (defined as the region between flanking SNPs) excluding the CO region from 2e, whose location could not be precisely determined. MEME was run with the following considerations: first, the spans vary widely in length, and we wanted to give more weight to motifs discovered in small spans than longer ones because the signal-to-noise ratio should be greater; second, we did not want to require MEME to find each motif in all sequences; third, because of the small number of sequences and MEME’s sensitivity to initial conditions, we wanted to ensure any candidate signal was found robustly.

To address these considerations we ran MEME with the “zoops” model (allowing zero-or-one motif instances per sequence) on 13 combinations of spans, starting with only the smallest 2 spans, then the smallest 3, working up to all 14 spans, and then looked for motifs found consistently in all runs. Motif width was capped at 20 bp and selected the top twenty motifs from each MEME run.

After motif discovery, we used FIMO (Grant *et al.* 2011) to search for motif instances in the input sequences. FIMO matches are biased toward longer motifs, which tend to have lower *P*-values. To partially address this we required that passing matches have (*P*-value × match length) ≥ 0.001.

To make an objective decision as to which motifs in which runs were equivalent, we used Tomtom (Gupta *et al.* 2007), which makes pairwise ungapped alignments of motifs and returns a *P*-value. With 13 runs at 20 motifs each, we aligned all 260 motifs to each other and thresholded the resulting connectivity matrix with the FIMO criterion of (*P*-value × alignment length) ≥ 0.001. After thresholding, all subgraphs were extracted from the connectivity matrix, and these became “metamotifs.” Each metamotif represents a pool of motifs that can be considered equivalent at the given level of stringency. As a test of significance for metamotifs, the same process was repeated on 100 sets of 14 randomly selected regions of chromosome X with the same size distribution as the CO spans. Relative enrichment for core motifs that contributed to a metamotif was performed by examining the distribution of core motifs within the 14 CO spans relative to the 100 random sets of 14 random sequence sets by comparing Observed/Expected ratios estimated based on nucleotide composition. Finally, local enrichment was examined because a motif that facilitates the formation of CO events might be significantly enriched in CO spans relative to the immediate flanking region and show a decrease in excess enrichment further from the CO site. We tested all 260 motifs for such enrichment using the Fisher’s exact test for CO spans *vs.* three background sets: 1 kb, 5 kb, and 10 kb on either side of the span.

## Results

Our experiment set out to precisely identify and quantify locations of CO and GC events in a single *Drosophila melanogaster* female meiosis. We approached this by first isogenizing two common lab strains, Canton-S and *w^1118^*, and sequencing them to determine the number of either SNPs or InDels that exist between the two lines. We then crossed the isogenized stocks to one another, allowed the females to undergo meiosis, and crossed resultant males to *C(1)DX* females. This preserved a nonrecombining clone of the original *X* in stock (see Figure S1 for cross scheme). This allowed us to maintain the isolated *X* chromosomes without recombination.

Illumina libraries were made from genomic DNA isolated from 10 males from either isogenized Canton-S and *w^1118^* stocks (parental lines) or from the resultant progeny of crosses to *C(1)DX* females (progeny lines). The parental libraries were run on two lanes, and the progeny libraries were run on a single lane of a flow cell on an Illumina Genome Analyzer IIx using 40 bp paired-end sequencing. Using this method, we obtained *X* chromosome coverage of 99% and an average read depth of 18.1x for the parental lines and an average coverage of 93% and an average read depth of 5.6x for the progeny lines (Table S1).

To identify SNPs and InDels, we independently compared each parental sequence to the reference *Drosophila* sequence (version 5.22). This generated a list of polymorphisms for each line that had a parental coverage of at least 4x and a consensus quality score of at least 30. We then compared the Canton-S and *w^1118^* sequences to each other, identifying polymorphisms that distinguished the two lines. This gave us 79,045 SNPs and 14,483 InDels for the two parental lines. Five more InDel polymorphisms were identified during Sanger verification of the intervals in which CO events occurred, resulting in a total of 93,528 total polymorphisms.

Using tools developed in-house, we performed SNP calling to identify haplotype identity along the hemizygous *X* chromosome. As with the parental lines, genotypes were called only at nucleotide positions with a depth of at least 4x and a MAQ consensus quality score of 30. Since male progeny were sequenced with less depth, approximately 70% of parental variants were called with this threshold in each of the progeny lines (Table S1). The approximately 30% remaining were not called with this stringency and excluded from analysis.

Initial analysis of 30 male progeny from four females identified 15 CO events and 28 putative GC events. All 15 CO events were verified with Sanger sequencing. Of 28 candidate GC events, verification using Sanger sequencing confirmed only five events, with 23 being false-positive results (Table S3 and Table S4). Closer analysis revealed that the 82% false-positive rate for GC events was largely the result of poor sequence alignment or InDels that were not identified during the variant calling in the parental lines.

### Location and positioning of COs

Fifteen COs were detected in 12 of the 30 progeny analyzed (Table 1). Nine chromosomes carried a single CO event, and three carried a DCO. None of the CO events displayed discontinuous tracts of GC. The presence of a CO event had no strong influence on the likelihood that a GC would be observed elsewhere on the chromosome arm (Fisher’s exact test, *P* = 0.63). To precisely define CO positions, primers were designed flanking each CO interval including the next neighboring polymorphism not defined by the CO event (Table S2). Verification of each CO event allowed us to narrow 14 of the events down to spans with an average length of 923 bp. The CO event from stock 2e at 9D3 was not precisely localized because the flanking SNPs identified in the parental lines were 18 kb apart. Verification of COs did not result in the discovery of new SNPs but did identify five new InDels not identified from the parental analysis. Many times these were poly-A regions or repeat-rich regions in which it is difficult to identify InDels based on the original WGS data. The five InDels found by Sanger sequencing that narrowed the boundary for a CO event were added to the total number of InDels found by whole genome sequencing and were included in the statistical analysis.

**Table 1:** CO span detail

Progeny	Class	Distal SNP	Parent	Proximal SNP	Parent	CO Span	Band	Location	Gene
2d	SCO	2,413,159	*w^1118^*	2,413,912	Canton-S	753	3A4	Intron	*trol*
3a	SCO	4,860,933	*w^1118^*	4,861,309	Canton-S	376	4E1	Intergenic	n/a
1e	DCO	5,441,397	Canton-S	5,441,591	*w^1118^*	194	5A4	Intron	*Vsx2*
1a	SCO	6,601,365	*w^1118^*	6,601,675	Canton-S	310	6C8	Intron	*CG14441*
1b	SCO	8,000,862	*w^1118^*	8,001,116	Canton-S	254	7D6	Intron	*Gclc*
2c	SCO	8,834,132	Canton-S	8,834,399	*w^1118^*	267	8C1	Intron	*rdgA*
3b	SCO	8,862,810	Canton-S	8,863,096	*w^1118^*	286	8C1	Intron	*rdgA*
2e	SCO	10,437,477	*w^1118^*	10,455,495	Canton-S	18,018	9D3	Intron, exon	*spri*
4a	DCO	11,968,504	Canton-S	11,969,790	*w^1118^*	1,286	11A3	Intergenic	n/a
2a	DCO	11,992,791	*w^1118^*	11,994,201	Canton-S	1,410	11A3	Intergenic	n/a
1c	SCO	12,813,592	*w^1118^*	12,814,988	Canton-S	1,396	11D3	Intergenic	n/a
1d	SCO	15,696,462	Canton-S	15,697,193	*w^1118^*	731	13F1	Intron, exon, and intergenic	*PGRP-LE*
1e	DCO	16,511,826	*w^1118^*	16,514,097	Canton-S	2,271	14F2	Intron	*CG9782*
4a	DCO	19,291,635	*w^1118^*	19,293,197	Canton-S	1,562	18C3	Intron	*kek5*
2a	DCO	19,451,465	Canton-S	19,453,294	*w^1118^*	1,829	18D1	Intergenic	n/a

All COs found in the progeny along with the SNP or InDel locations used to define the boundaries of the CO. Progeny indicates the individual F1 identifier. Number indicates mother and letter designates specific progeny of a given mother. Distal SNP indicates the position of most distal SNP flanking the crossover. Parent indicates the genotypic designation of the SNP. Proximal SNP indicates the position of most proximal SNP flanking the crossover. Parent indicates the genotypic designation of the SNP. CO span indicates the distance between flanking SNPs. Band indicates the cytological position of crossover. Location indicates the identity of crossover position. Finally, gene indicates the gene identifier for crossovers in introns or exons.

CO, crossover; SNP, single-nucleotide polymorphism; SCO, single crossover; DCO, double crossover.

**Figure 1:**
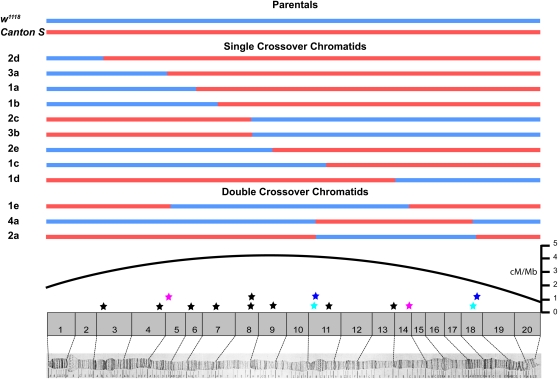
Parental haplotype structure and the distribution of COs. Of 30 chromosomes recovered, nine experienced one CO and three experience two COs. Pairs of colored stars indicate DCO positions. For reference, the per Mb recombination rate is provided (Fiston-Lavier *et al.* 2010).

All 9 SCO chromatids carried CO events that were distributed over the distal two-thirds of the *X* chromosome (Figure 1). Indeed, seven SCOs were located in the distal-most half of the *X* chromosome. The average pairwise distance between these single COs was 4.8 Mb. The three observed DCO chromatids each carry two single CO events separated by an average distance of 8.6 Mb. The greater distance between CO events on the DCO chromatids indicates the presence of CO interference. Overall, the distribution of COs along the arm of the *X* was as expected by previous genetic studies (Lindsley and Sandler 1977).

Of the 14 CO regions placed within a span of flanking SNPs, eight of these spanned entirely intronic sequence, five entirely spanned intergenic sequence, and one spanned a mixture of exonic, intronic, and intergenic sequence (Table 1). The total sum of these CO spans comprised 12,925 bp, of which 426 bp was exonic sequence, yielding the proportion of CO spans comprised of exonic sequence equal to 3.3%. This result is in contrast to the entire *X* chromosome, which comprises 23.7% exonic sequence. To test for significance, we generated 1000 random datasets with an equal size distribution of spans and estimated the proportion of exonic sequence within each of the random sets. The average percent of exonic sequence in these trials was 23.4%, and only 15 trials produced a percent exonic sequence less than or equal to 3.3%, yielding an empirical *P*-value of 0.015. These results may indicate that CO events in *Drosophila melanogaster* have a tendency to avoid exonic regions. This is a conservative conclusion, in light of the fact that intron length is lower in regions with a greater rate of crossing over (Carvalho and Clark 1999; Comeron and Kreitman 2000) and one might therefore expect COs to be more likely to contain exonic sequence than at random.

Interestingly, two independent CO events from different mothers occurred within the same intron of the same gene, *rdgA* (Table 1). The COs were minimally 28,411 kb and maximally 28,964 kb apart and occurred in the third intron of the most common transcript of the *rdgA* gene, which is approximately 94 kb long.

### CO events are not significantly clustered in 8C1, 11A3, and 18C/D

Although our sample of 15 COs was small, we nonetheless found three regions (8C1, 11A3, and 18C3-18D1) in which two independent CO events were detected within the same small cytological interval. In two of the three locations, the COs were in the same cytological band (8C1 and 11A3) and were approximately 28.5 kb apart, and in the third case (18C3/18D1), the COs were approximately 158 kb apart (Figure 1). Although it may seem unusual to observe events only 28.5 kb apart on a 22.4-Mb chromosome, a numerical simulation (see *Materials and Methods*) showed that given a set of 15 events, observing the closest pair of two CO events occurring within a distance of 25 kb was to be expected more than 30% of the time.

To further investigate the possibility that the frequency of meiotic recombination might be elevated in regions 8C1, 11A3, and 18C3-18D1, we generated an additional 96 recombinant chromosomes by repeating the cross described in Figure S1. These 96 chromosomes were then assayed for recombination within the three intervals by restriction digest genotyping of SNPs flanking each interval. Only one recombination event, between 10F3 and 11A7, was observed within the 257 tested intervals, a result that is inconsistent with substantially elevated recombination rates in these regions. We conclude that the observation of three relatively close pairs of events in our initial sample of 15 was most likely the result of chance, but note that this experiment had low power to detect recombination rate heterogeneity.

### CO motif analysis

To identify potential DNA sequence motifs associated with sites of crossing over, we used the MEME software package (Bailey and Elkan 1995a, 1995b; Bailey *et al.* 2006). We restricted our analysis to CO spans because potential mechanistic differences between CO and non-CO events could potentially add noise to an aggregate discovery approach. Our analysis also excluded the one 18-kb minimal CO span from male stock 2e, as the signal-to-noise ratio would be substantially increased by its inclusion. This left a sample of 14 CO spans to search for motifs. Because MEME results can be somewhat sensitive to modest variation in nucleotide composition among different domains, we used an iterative procedure that varied the number of regions analyzed per run. Specifically, we performed 13 different MEME runs, the first of which contained the two smallest (and potentially most informative) CO spans, and then added the next largest CO span to each subsequent run.

Each of these 13 MEME runs identified the top 20 motifs that were more common than expected on the basis of nucleotide composition. To identify shared features across motifs, a metamotif analysis was performed by aligning the best 20 motifs discovered from each MEME run. Three metamotifs with specific features were identified. The metamotif with the largest number of member motifs identified (MM1, not shown), comprised several poly-A stretches and was found in eight of 13 MEME runs. Such low complexity homo-polymers are statistically unexpected on the basis of single-base nucleotide composition but are obviously quite common in the genome.

**Figure 2:**
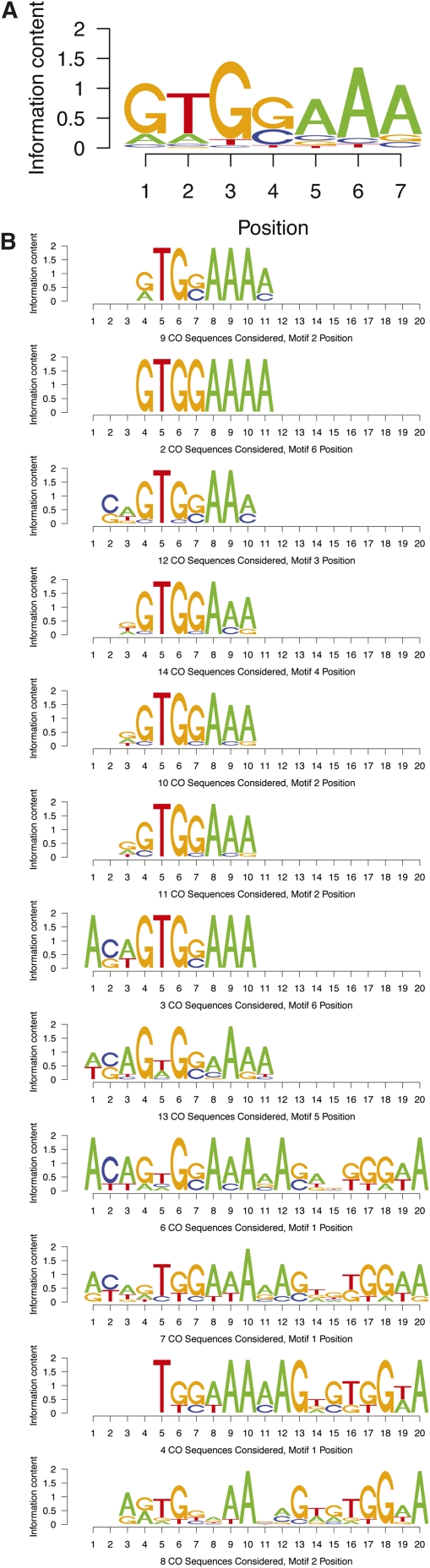
CO motif analysis. (A) Metamotif2 (MM2) summary logo. Multiple alignment of the MM2 member motifs, one each from 12/13 MEME runs, derived from pairwise alignments by Tomtom. (B) Similar member motifs contributing to MM2, identified from 12/13 different MEME runs, using iterative addition of CO regions.

The second largest metamotif (MM2) (Figure 2a) contained several variants on a core GTGGAAA sequence and was identified in 12 of 13 MEME runs (Figure 2b). Finding a sequence of this length is unexpected and the fact that 12 runs identified this common core is consistent with this motif being biologically relevant. The third largest metamotif (MM3, not shown) was only recovered from seven of 13 MEME runs; thus, it did not represent a sufficiently strong signal; smaller metamotifs were even less well represented. Therefore, we concluded that MM2 appeared to be the most promising candidate for a CO signal. No similarity to known transcription factor binding sites was identified for this motif in either the TRANSFAC (Matys *et al.* 2006) or JASPAR (Portales-Casamar *et al.* 2010) databases.

To further assess the significance of MM2, several statistical analyses were performed. First we determined whether the core sequences that comprise the motif were enriched in CO spans relative to the entire chromosome. Using just the core GTGGAAA sequence, we found that this sequence is enriched in CO regions by a factor of 5.9 relative to the rest of chromosome *X* (P-value *vs.* 100 random sets: 5.23E-03). If the CO signal is genuine, then, we might also assume that the core GTGGAAA sequence is significantly enriched in the CO span *vs.* the immediate flanking region and that the enrichment will decrease as the flanking regions widen, as the motif abundance begins to equalize. We tested motif enrichment in CO spans *vs.* 1 kb, 5 kb, and 10 kb left and right flanking sequences using Fisher’s exact test. As not to be biased toward members of MM2, we tested all 260 CO motifs in this manner.

For enrichments relative to 1-kb flanks, only 11 of 260 motifs were significant after Benjamini-Hochberg correction (adjusted *P* ≤ 0.05): all were overenriched, and none was significant at 5 kb or 10 kb (although one had adjusted *P* = 0.057 at 5 kb). Three of these motifs were from MM2, the largest representation for any metamotif; no other significant metamotifs had enriched members. At adjusted *P* < = 0.01, one-half of the significant motifs were contributed by these three motifs that contributed to MM2. We thus conclude that this sequence is enriched in CO regions relative to 1-kb flanking regions, suggesting that local domains of enrichment can facilitate local positioning of CO events.

Second, to estimate the likelihood of finding a motif of equal complexity to MM2 by chance, we generated 100 sets of 14 randomly selected *X* chromosome sequences as long as the original 14 CO regions and repeated our iterative motif search analysis. If MM2 is a false-positive result, then repeating our MEME analysis on random sequences would be expected to identify similarly complex motifs quite often. Of these 100 simulated sets of 14 regions, only two sets yielded a metamotif that showed up in at least 12 of 13 MEME runs and also had as strong an e-value and information score. This yields an empirically derived *P*-value of 0.02 for MM2. Finally, given the previous observation that COs were enriched in intronic and intergenic regions, we examined the distribution of the three core components of MM2 (GTGGAAA, GTGCAAA, and ATGGAAA) in these regions. Intronic and intergenic regions comprise 76% of the X chromosome and contain 86% of these motifs located on the X. Thus, MM2 is enriched in intronic and intergenic regions on the X.

### Analysis of GC

**Figure 3:**
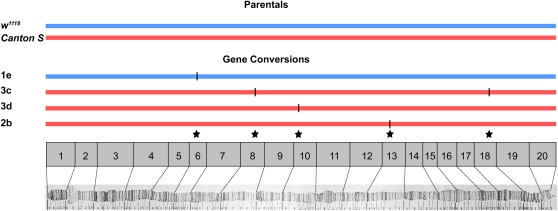
Parental haplotype structure and the distribution of non-CO GC events. Five events were identified in four progeny.

We observed five GC events (Figure 3). GC events appear to be more evenly distributed along the entire euchromatic arm of the *X* chromosome than are CO events, although a lack of statistical power arising from only recovering five GC events precludes a rigorous test of this hypothesis. That said, the distribution of GC events does appear to be different from the distribution of CO events, all of which occurred over the distal two-thirds of the *X* chromosome.

We defined GC tracts on the basis of their maximum possible size—the entire span between nonconverted SNPs. Of the five GC tracts placed within a span of flanking SNPs, one was entirely intronic, two entirely intergenic, and two contained an intron and exon. In aggregate, these tracts comprised 8151 bp of sequence, of which 1845 bp were exonic. This yields a total proportion of exonic sequence in GC regions being equal to 22.6% (Table 2) . This finding is similar to the proportion of the *X* chromosome comprised of exonic sequence (23.7%) and stands in contrast to CO spans, for which only one of 14 regions contained an exon, comprising 3.3% of total sequence. To test the significance, we generated 1000 random data sets equivalent to the distribution of localized GC tracts and determined the frequency for which the proportion of exonic sequence was equal to or less than 3.3%. This experiment yielded a *P*-value of 0.096, significant only at the 0.1 level. Thus, it appears that GCs are uniformly distributed across exons, but with a sample size of five GC events, we lack power in our test for a difference in the distribution of GC and CO events.

**Table 2:** GC tract detail

Progeny	Distal SNP	First SNP	SNP Count	Last SNP	SNPs From	Proximal SNP	GC Tract Length (min−max)	Band	Location	Gene
1e	6,633,119	6,633,448	4	6,633,592	Canton-S	6,633,865	144−746	6C10	Intron, exon	*CG3168*
3c	9,157,653	9,159,700	3	9,159,935	*w^1118^*	9,161,089	235−3,436	8D4	Intron, exon	*CG32703*
3d	10,914,005	10,914,112	15	10,914,724	*w^1118^*	10,916,406	612−2,401	10A2	Intron	*CG42339*
2b	15,151,729	15,151,810	3	15,151,997	*w^1118^*	15,152,643	187−914	13B4	Intergenic	n/a
3c	19,520,853	19,521,068	3	19,521,155	*w^1118^*	19,521,507	87−654	18D8	Intergenic	n/a

All GCs found in the progeny along with the SNP locations used to define the boundaries. Progeny indicates individual F1 identifier. Number indicates mother and letter designates specific progeny of a given mother. Distal SNP indicates the position of most distal nonconverted SNP flanking the gene conversion. First SNP indicates the position of most distal converted SNP. SNP count indicates the number of SNPs converted within tract. Last SNP indicates the position of most proximal converted SNP. “SNPs from” indicates the genotypic designation of converted SNPs. Proximal SNP indicates the position of most proximal nonconverted SNP flanking the gene conversion. GC Tract Length (min-max) indicates the minimum and maximum tract length. Minimum tract length is distance between converted SNPs. Maximum tract length is distance between nonconverted SNPs. Band indicates the cytological position of crossover. Location indicates the identity of gene conversion position. Finally, gene indicates the ene identifier for conversions in introns or exons.

GC, gene conversion; SNP, single-nucleotide polymorphism.

**Figure 4:**
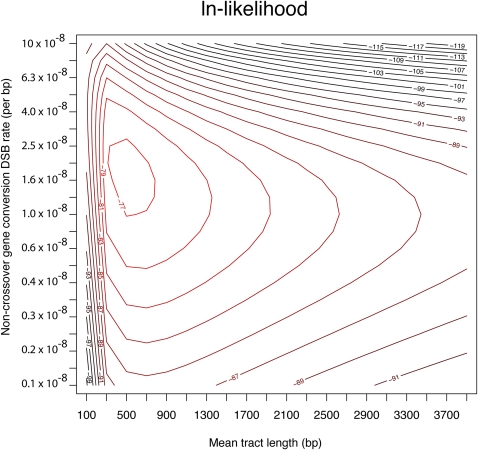
Ln-likelihood surface for joint estimation of the rate of DSB formation for non-CO GC events and the GC tract length. Non-CO GC events are the events that arise from DSBs designated as non-CO GC events. The rate of these events is given per bp per meiosis. This differs from the GC rate, which is the rate at which nucleotides become converted. The GC rate is the multiple of the non-CO GC DSB rate and the GC tract length.

Although all COs are visible by this sequencing method because of the easy detection of exchange of flanking markers, GCs are invisible if they occur entirely between SNP markers. Therefore, we developed a maximum likelihood method to jointly estimate the GC frequency and tract length (Figure 4). The basic principle of this method is to estimate these two parameters considering the five visible GC events, as well as the distribution of distances between SNPs for which no GC event was observed. From this analysis, the joint ML estimate for the rate of GC event occurrence was 1.8 × 10^−8^ per bp (95% CI 0.6 × 10^−8^ to 3.9 × 10^−8^) and average one-sided tract length was 238 bp (95% CI 117−543) This yields a total mean GC tract length of 476 bp, which is similar to other estimates of mean tract length estimated from the *rosy* locus, ranging from 352 bp (Hilliker *et al.* 1994) to 441 bp (Blanton *et al.* 2005). Considering the rate of GC event occurrence and mean tract length, our data yield a per bp GC rate of 0.86 × 10^−5^, which is similar to previous estimates of 1.3 × 10^−5^ based on genetic analysis of the *rosy* locus (Blanton *et al.* 2005). As the assembled *X* chromosome scaffold is 22.4 × 10^6^ bp, the per-chromosome arm rate of GC event occurrence is 0.40. This rate would predict 12 expected GC events across 30 *X* chromosomes. Because only five GC events were directly observed among thirty F1 progeny (16%), this finding indicates that more than one-half of the GC events were likely missed because of the fact that the conversion tract did not include a genotyped SNP marker.

By applying the 1.8 × 10^−8^ GC events per bp rate to the entire genome (119 Mb, excluding the fourth chromosome and heterochromatin), the genome wide rate of euchromatic GC is estimated to be 2.14 GC events per haploid meiotic product. Because meiosis results in four haploid products, this implies 8.6 GC events per meiosis. Considering that 5 COs are estimated to occur per meiosis (Carpenter 1975), this yields a GC:CO ratio of 1.7:1. This assumes GC is an entirely asymmetrical process in which only the chromatid that has experienced the DSB receives the converted genetic information.

Overall, we conclude there are approximately 13.6 euchromatic recombination events per meiosis. Previous studies have found there are approximately 21 DSBs per meiosis in *D. melanogaster*, and these appear to be outside of the heterochromatin (Jang *et al.* 2003; Klovstad *et al.* 2008; Mehrotra and McKim 2006). If heteroduplex repair always results in GC, our estimate of euchromatic DSBs is therefore 13.6. To test whether this is significantly different than the number of DSBs measured previously by cytology, we performed a likelihood-ratio test to conservatively determine whether our estimate would be inconsistent with a model of 20 DSBs which are repaired as either CO or GC events. We are unable to reject 20 recombination events (*P* = 0.15). Moreover, if one-half of the formed heteroduplex structures are repaired without GC, our estimate for non-CO DSBs would be twice our 8.6 estimate. In total, this would yield 22 DSBs, quite similar to previous estimates. Considering a low estimate of 13.5 recombination events per meiosis, these results are consistent with a modest number of meiotic DSBs in *D. melanogaster* being repaired by other mechanisms, such as sister chromatid events (Goldfarb and Lichten 2010), but it is not conclusive.

### GC motif analysis

With only five GC events, we had little power to search specifically for motifs enriched in GC tracts. Instead, we examined the distribution of previously described CO motifs in the GC tracts. Focusing on the 260 CO motifs identified from the 13 MEME runs, we tested for enrichment in the GC tracts. Relative to 1-kb flanks, only three were significant, with *P*-values uncorrected for multiple tests and none after correction. Many were significant *vs.* 10-kb flanks and without exception these were all underenriched, indicating the uniqueness of the CO *vs.* GC regions.

In addition, we sought to compare the representation of the three strongest contributors to MM2, GTGGAAA, GTGCAAA, and ATGGAAA, between CO and GC regions. In CO spans, summing to a total of 12,925 bp, these occurred 10, eight, and seven times respectively. In GC tracts, summing to a total of 8,151 bp, these occurred two, two, and three times respectively. By a Fisher’s exact test considering all possible nonoverlapping, seven bp positions, *P* = 0.038. This result indicates that the core motifs that contribute to MM2 are significantly more enriched in CO spans relative to GC tracts.

## Discussion

Meiotic recombination plays a key role in two fundamental aspects of biology—maintaining proper chromosome segregation in the production of gametes and allowing the evolutionary fate of alleles to become unhindered by linkage. Although many advances have been made in dissecting the machinery of meiotic recombination, very little is known about how the landscape of meiotic recombination is determined across the genome. Across all eukaryotes, the formation of DSBs is a key initiating factor of recombination (Dernburg *et al.* 1998; Grelon *et al.* 2001; McKim and Hayashi-Hagihara 1998; Sun *et al.* 1989). Thus, the rate and landscape of meiotic recombination fundamentally depends on DSB formation, but how DSBs are established is poorly understood. How the fate of these DSBs is determined—either through crossing over or through other forms of repair—is also poorly understood. This “fate decision” is crucial in gamete formation because crossing over plays an important role in proper chromosome segregation. From an evolutionary perspective, this fate also plays a key role in how patterns of LD are determined across the genome since GC breaks up LD only across shorter distances. A key to understanding the factors that determine the landscape of recombination is a determination of the chromosome-wide distribution of recombination events at the greatest resolution possible. New sequencing technologies now make this possible.

Using a WGS approach, we have obtained the first high-resolution view of the recombination landscape across a chromosome in *Drosophila*. This approach allows a level of analysis of recombination that has not been previously available. In particular, it allows one to jointly estimate the overall rate of CO and GC as well as determine the precise location and form of recombination events without restricting analysis to a single locus. Comfortingly, this study confirms nearly a century’s worth of *Drosophila* genetics using an entirely different approach. In particular, our estimates for rate and distribution of crossing over are very similar to those using standard approaches. Likewise, using a statistical approach that jointly estimates GC frequency and GC tract length allows us to reconcile cytological studies of DSB formation with genetic studies of GC at the *rosy* locus. In particular, our chromosome-wide estimate of GC rate is close to that estimated from the *rosy* locus. Considering both COs and GCs, our lower estimate of DSBs that become repaired through recombination (13.6) is similar to, but less than, previous estimates of total meiotic DSBs (~21). This finding supports the observation that other mechanisms, such as sister chromatid repair as recently seen in yeast (Goldfarb and Lichten 2010), may also contribute to meiotic DSB repair. Further studies in a genetic background with more precisely known meiotic DSB numbers will be necessary to formally test this hypothesis.

Aside from rate estimates for CO and GC that are consistent with many previous studies, we also find that the structure of CO and GC events is similar to that found in previous studies. In particular, we find no evidence for discontinuous tracts of GC for either CO or NCO GC. This is finding consistent with studies of the *rosy* locus in wild-type flies that show the great majority of GC tracts are continuous (Carpenter 1982; Curtis and Bender 1991; Curtis *et al.* 1989; Radford *et al.* 2007a, 2007b). Moreover, our estimate for GC tract length—a total mean GC tract length of 476 bp—is similar to other estimates of mean tract length estimated from the *rosy* locus, ranging from 352 bp (Hilliker *et al.* 1994) to 441 bp (Blanton *et al.* 2005). Overall, this analysis suggests that the *rosy* locus serves as an excellent model for studying the mechanisms of recombination.

Although these results have largely confirmed previous studies, our ability to precisely define the location of recombination events across the *X* has provided novel insights. This has particular significance in explaining the mechanisms that determine fine-scale patterns of heterogeneity in the recombination rate. One striking finding is that domains of crossing over tend to avoid exonic regions. This may be mediated by chromatin marks that are enriched on exons in a manner similar to that observed in other species (Dhami *et al.* 2010; Kolasinska-Zwierz *et al.* 2009). It is also reminiscent of the observation in humans that recombination preferentially occurs outside of genes and exons (Kong *et al.* 2010; McVean *et al.* 2004; Myers *et al.* 2005). In addition, we have identified a short sequence motif (GTGGAAA) that is enriched in CO spans. This differs from previously identified motifs that correlate with overall recombination rate in *D. pseudoobscura* (Kulathinal *et al.* 2008) or *D. persimilis* (Stevison and Noor 2010) *i.e.* CCCCACCCC, CCTCCT, CACAC, ATAAA, and AATAA. It also differs from the greater complexity motifs identified in mammalian systems but not found in our analysis, such as CCNCCNTNNCCNC in humans, that are associated with CO hotspots (Myers *et al.* 2008) and are also a predictor of recombination rate in *D. persimilis* (Stevison and Noor 2010)

At first glance, it might not be clear why our motif results would appear different from those found in *D. pseudoobscura* and *D. persimilis*. However, the experiments performed in *D. pseudoobscura* and *D. persimilis* were different; substantially more meiotic events were analyzed (>1000) but with fewer markers distributed along the chromosome arm. Thus, those studies identified a substantially greater number of recombination events at lower resolution, whereas this study examined substantially fewer CO sites at greater precision. By investigating a large number of meiotic events, Stevison and Noor (2010) were able to examine broad correlations between overall recombination rate and overall genome content whereas we were unable to do so. Instead, we examined the distribution of sequences within CO and GC regions that were precisely localized. In addition, Stevison and Noor examined *Drosophila* species that have diverged substantially from *D. melanogaster*. Given the differences in the sequence composition of recombinational hotspots already present among humans and chimpanzees (Hinch *et al.* 2011; Myers *et al.* 2010), different recombination motifs may have evolved in these two species compared with *D. melanogaster*.

Despite finding only five GC events, their precise localization may provide at least some insight into the mechanisms of how DSBs are destined to be repaired as either COs or NCOs. In particular, the two factors that we found significant determinants of the precise localization of CO events—the avoidance of exons and the enrichment of MM2—do not appear to apply to GC events (with the caveat that there is not a substantial amount of statistical power in our sample). The proportion of GC tracts composed of exonic sequence is nearly the same as the background exonic composition of the *X*, albeit their enrichment is significantly different from CO regions only at the 0.1 level. We also find none of the motifs that are enriched in CO spans are enriched in GC tracts relative to flanking regions and there is greater enrichment of the three core motifs that comprise MM2 in CO spans relative to GC tracts.

There are currently two models for how the CO/NCO choice is made among DSBs. In one model, after the occurrence of a DSB, a double Holliday Junction (dHJ) forms, and CO/NCO choice is determined on the basis of the resolution of the dHJ (Szostak *et al.* 1983). A more recent and well-supported model, however, suggests the CO/NCO designation is achieved before the formation of dHJ, and the NCOs occur through DSB repair without dHJ formation (Berchowitz and Copenhaver 2010; Bishop and Zickler 2004; Youds and Boulton 2011). According to each of these models, CO/NCO choice occurs downstream of DSB formation. In light of these two models, these results suggest that these properties of the DNA at the site of the DSB influence CO/NCO designation, rather than the formation of a DSB *per se*. If our identified sequence features solely mediated DSB formation, we would expect to see them in CO and GC regions alike. Thus, subsequent to DSB formation, the presence of MM2 and the absence of exonic sequence may encourage CO formation. An alternative model, in which CO/NCO designation is made during or before the formation of DSB, is also possible. In this case, the presence of the MM2 motif and the absence of exonic sequence would facilitate the formation of DSBs that are destined for CO and DSBs that occur independent of these sequence characteristics would be more likely to result in NCOs. In this case, the absence of *Drosophila* CO hotpots might be explained by the fact that both nonexonic sequence and the simple MM2 motif are both broadly distributed across the *X* chromosome and thus not sufficiently localized to drive DSB formation in a manner that would have by now been detected. Further high-resolution studies will be required to test these models and also include the influence of interference.

Overall, we have demonstrated that sequencing progeny from a single round of meiosis by WGS will prove to be a powerful method in dissecting the mechanisms of meiotic recombination. In our study, we focused only on the *X* chromosome for several reasons. By using a *C(1)DX* stock, we were able to “clone” single recombinant *X* chromosomes without being confounded by GC events that may subsequently occur over balancer chromosomes. A second significant reason for focusing on the *X* arose from the costs related to obtaining very high levels of sequence coverage necessary for the study of recombination on autosomes. Identifying recombination events on the *X* in F1 males requires simply identifying the haplotype structure along a single hemizygous chromosome. In contrast, the scoring of recombination events on the autosomes of F1 progeny requires being able to distinguish heterozygosity from homozygosity. In the case of GC, one would in fact be required to distinguish heterozygosity and homozygosity for every nucleotide polymorphic between the parents. This becomes very challenging without great sequencing depth and would require extensive downstream verification. We also randomly selected male progeny to create *C(1)DX*-bearing stocks for sequencing rather than selecting males know to carry recombinant *X* chromosomes. We did this so as to not bias our estimates of the rate of GC if the rate of COs or GCs were dependent. Our results suggest that the likelihood of a GC event on a chromosome was not influenced by a CO event, though we lacked power for this test.

Of additional significance, we see this technique as the way meiotic mutants will be analyzed in the future. The rapidly decreasing cost of sequencing and the increase in data provided with each experiment is approaching a point where it is cheaper to analyze individual genomes than it is to do traditional crosses when analyzing meiotic mutants. Most importantly, very few meiotic mutants have been assessed for their effects on GC (Blanton *et al.* 2005; Carpenter 1982; Curtis and Bender 1991; Radford *et al.* 2007a). This missing knowledge is especially problematic for the analysis of those mutants that affect pairing and/or synapsis as *c(3)G*, *cona*, *c(2)M*, and *ord* as well as mutants likely to come out of ongoing screens.

In summary, we provide the first step toward a WGS-based approach to the study of meiotic recombination in *D. melanogaster*. Although the step was admittedly a small one, even these limited data point to curious differences in distribution of CO and GC events, a bias against COs occurring in exonic regions, and a motif enriched in the vicinity of CO events. As technologies improve and costs continue to decrease, we expect that these inferences will be rigorously tested and the analysis extended to meiotic mutants. Indeed, we look forward to a day in the not-so-distant future when characterizing the recombination landscape with visible markers becomes a practice primarily discussed in undergraduate lecture courses.

## Supplementary Material

Supporting Information

## References

[bib1] Bailey T. L. Elkan C., 1994 Fitting a mixture model by expectation maximization to discover motifs in biopolymers. Proc. Int. Conf. Intell. Syst. Mol. Biol. 2: 28–36 7584402

[bib2] Bailey T. L. Elkan C., 1995 a The value of prior knowledge in discovering motifs with MEME. Proc. Int. Conf. Intell. Syst. Mol. Biol. 3: 21–29 7584439

[bib3] Bailey T. L. Elkan C., 1995 b Unsupervised learning of multiple motifs in biopolymers using expectation maximization. Machine Learning Journal 21: 51–83

[bib4] Bailey T. L. Williams N. Misleh C. Li W. W., 2006 MEME: discovering and analyzing DNA and protein sequence motifs. Nucleic Acids Res. 34: W369–W373 16845028 10.1093/nar/gkl198 PMC1538909

[bib5] Berchowitz L. E. Copenhaver G. P., 2010 Genetic interference: don’t stand so close to me. Curr. Genomics 11: 91–102 20885817 10.2174/138920210790886835 PMC2874225

[bib6] Bishop D. K. Zickler D., 2004 Early decision: meiotic crossover interference prior to stable strand exchange and synapsis. Cell 117: 9–15 15066278 10.1016/s0092-8674(04)00297-1

[bib7] Blanton H. L. Radford S. J. McMahan S. Kearney H. M. Ibrahim J. G., 2005 REC, Drosophila MCM8, drives formation of meiotic crossovers. PLoS Genet. 1: 343–354 10.1371/journal.pgen.0010040 PMC1231718 16189551

[bib8] Blumenstiel J. P. Noll A. C. Griffiths J. A. Perera A. G. Walton K. N., 2009 Identification of EMS-induced mutations in *Drosophila melanogaster* by whole-genome sequencing. Genetics 182: 25–32 19307605 10.1534/genetics.109.101998 PMC2674820

[bib9] Carpenter A. T. C., 1975 Electron microscopy of meiosis in *Drosophila melanogaster* females. 2. Recombination nodule - recombination-associated structure at pachytene. Proc. Natl. Acad. Sci. USA 72: 3186–3189 810799 10.1073/pnas.72.8.3186 PMC432946

[bib10] Carpenter A. T. C., 1982 Mismatch repair, gene conversion, and crossing-over in two recombination-defective mutants of *Drosophila melanogaster*. Proc. Natl. Acad. Sci. U S A 79: 5961–5965 6821126 10.1073/pnas.79.19.5961 PMC347031

[bib11] Carvalho A. B. Clark A. G., 1999 Intron size and natural selection. Nature 401: 344 10517631 10.1038/43827

[bib12] Chovnick A. Ballantyne G. H. Holm D. G., 1971 Studies on gene conversion and its relationship to linked exchange in Drosophila melanogaster. Genetics 69: 179–209 5002749 10.1093/genetics/69.2.179 PMC1212696

[bib13] Comeron J. M. Kreitman M., 2000 The correlation between intron length and recombination in drosophila. Dynamic equilibrium between mutational and selective forces. Genetics 156: 1175–1190 11063693 10.1093/genetics/156.3.1175 PMC1461334

[bib14] Curtis D. Bender W., 1991 Gene conversion in Drosophila and the effects of the meiotic mutants mei-9 and mei-218. Genetics 127: 739–746 2029970 10.1093/genetics/127.4.739 PMC1204401

[bib15] Curtis D. Clark S. H. Chovnick A. Bender W., 1989 Molecular analysis of recombination events in Drosophila. Genetics 122: 653–661 2503419 10.1093/genetics/122.3.653 PMC1203738

[bib16] DePristo M. A. Banks E. Poplin R. Garimella K. V. Maguire J. R., 2011 A framework for variation discovery and genotyping using next-generation DNA sequencing data. Nat. Genet. 43: 491–498 21478889 10.1038/ng.806 PMC3083463

[bib17] Dernburg A. F. McDonald K. Moulder G. Barstead R. Dresser M., 1998 Meiotic recombination in *C. elegans* initiates by a conserved mechanism and is dispensable for homologous chromosome synapsis. Cell 94: 387–398 9708740 10.1016/s0092-8674(00)81481-6

[bib18] Dhami P. Saffrey P. Bruce A. W. Dillon S. C. Chiang K., 2010 Complex exon-intron marking by histone modifications is not determined solely by nucleosome distribution. PLoS ONE 5: e12339 20808788 10.1371/journal.pone.0012339 PMC2925886

[bib19] Finnerty V. G., 1976 Gene conversion in *Drosophila*, pp. 331–351 in The Genetics and Biology of Drosophila, edited by Ashburner M. Novitski E. Academic Press, London

[bib20] Fiston-Lavier A. S. Singh N. D. Lipatov M. Petrov D. A., 2010 *Drosophila melanogaster* recombination rate calculator. Gene 463: 18–20 20452408 10.1016/j.gene.2010.04.015

[bib21] Goldfarb T. Lichten M., 2010 Frequent and efficient use of the sister chromatid for DNA double-strand break repair during budding yeast meiosis. PLoS Biol. 8: e1000520 20976044 10.1371/journal.pbio.1000520 PMC2957403

[bib22] Grant C. E. Bailey T. L. Noble W. S., 2011 FIMO: scanning for occurrences of a given motif. Bioinformatics 27: 1017–1018 21330290 10.1093/bioinformatics/btr064 PMC3065696

[bib23] Green M. M. Green K. C., 1949 Crossing-over between alleles at the lozenge locus in *Drosophila melanogaster*. Proc. Natl. Acad. Sci. U S A 35: 586–591 15391347 10.1073/pnas.35.10.586 PMC1063087

[bib24] Grelon M. Vezon D. Gendrot G. Pelletier G., 2001 AtSPO11-1 is necessary for efficient meiotic recombination in plants. EMBO J. 20: 589–600 11157765 10.1093/emboj/20.3.589 PMC133473

[bib25] Gupta S. Stamatoyannopoulos J. A. Bailey T. L. Noble W. S., 2007 Quantifying similarity between motifs. Genome Biol. 8: R24 17324271 10.1186/gb-2007-8-2-r24 PMC1852410

[bib26] Hey J. Kliman R. M., 2002 Interactions between natural selection, recombination and gene density in the genes of Drosophila. Genetics 160: 595–608 11861564 10.1093/genetics/160.2.595 PMC1461979

[bib27] Hilliker A. J. Harauz G. Reaume A. G. Gray M. Clark S. H., 1994 Meiotic gene conversion tract length distribution within the *rosy* locus of *Drosophila melanogaster*. Genetics 137: 1019–1024 7982556 10.1093/genetics/137.4.1019 PMC1206049

[bib28] Hinch A. G. Tandon A. Patterson N. Song Y. Rohland N., 2011 The landscape of recombination in African Americans. Nature 476: 170–175 21775986 10.1038/nature10336 PMC3154982

[bib29] Jang J. K. Sherizen D. E. Bhagat R. Manheim E. A. McKim K. S., 2003 Relationship of DNA double-strand breaks to synapsis in Drosophila. J. Cell Sci. 116: 3069–3077 12799415 10.1242/jcs.00614

[bib30] Klovstad M. Abdu U. Schupbach T., 2008 Drosophila brca2 is required for mitotic and meiotic DNA repair and efficient activation of the meiotic recombination checkpoint. PLoS Genet. 4: e31 18266476 10.1371/journal.pgen.0040031 PMC2233675

[bib31] Kolasinska-Zwierz P. Down T. Latorre I. Liu T. Liu X. S., 2009 Differential chromatin marking of introns and expressed exons by H3K36me3. Nat. Genet. 41: 376–381 19182803 10.1038/ng.322 PMC2648722

[bib32] Kong A. Gudbjartsson D. F. Sainz J. Jonsdottir G. M. Gudjonsson S. A., 2002 A high-resolution recombination map of the human genome. Nat. Genet. 31: 241–247 12053178 10.1038/ng917

[bib33] Kong A. Thorleifsson G. Gudbjartsson D. F. Masson G. Sigurdsson A., 2010 Fine-scale recombination rate differences between sexes, populations and individuals. Nature 467: 1099–1103 20981099 10.1038/nature09525

[bib34] Kulathinal R. J. Bennettt S. M. Fitzpatrick C. L. Noor M. A. F., 2008 Fine-scale mapping of recombination rate in Drosophila refines its correlation to diversity and divergence. Proc. Natl. Acad. Sci. U S A 105: 10051–10056 18621713 10.1073/pnas.0801848105 PMC2481358

[bib35] Langley C. H. Crepeau M. Cardeno C. Corbett-Detig R. Stevens K., 2011 Circumventing heterozygosity: sequencing the amplified genome of a single haploid drosophila melanogster embryo. Genetics 188: 239–246 21441209 10.1534/genetics.111.127530 PMC3122310

[bib36] Li H. Ruan J. Durbin R., 2008 Mapping short DNA sequencing reads and calling variants using mapping quality scores. Genome Res. 18: 1851–1858 18714091 10.1101/gr.078212.108 PMC2577856

[bib37] Li H. Handsaker B. Wysoker A. Fennell T. Ruan J., 2009 The Sequence Alignment/Map format and SAMtools. Bioinformatics 25: 2078–2079 19505943 10.1093/bioinformatics/btp352 PMC2723002

[bib38] Lindsley D. L. Sandler L., 1977 The genetic analysis of meiosis in female *Drosophila melanogaster*. Philos. Trans. R. Soc. Lond. B Biol. Sci. 277: 295–312 16292 10.1098/rstb.1977.0019

[bib39] Mancera E. Bourgon R. Brozzi A. Huber W. Steinmetz L. M., 2008 High-resolution mapping of meiotic crossovers and non-crossovers in yeast. Nature 454: 479–485 18615017 10.1038/nature07135 PMC2780006

[bib40] Matys V. Kel-Margoulis O. V. Fricke E. Liebich I. Land S., 2006 TRANSFAC and its module TRANSCompel: transcriptional gene regulation in eukaryotes. Nucleic Acids Res. 34: D108–D110 16381825 10.1093/nar/gkj143 PMC1347505

[bib41] McKenna A. Hanna M. Banks E. Sivachenko A. Cibulskis K., 2010 The Genome Analysis Toolkit: a MapReduce framework for analyzing next-generation DNA sequencing data. Genome Res. 20: 1297–1303 20644199 10.1101/gr.107524.110 PMC2928508

[bib42] McKim K. S. Hayashi-Hagihara A., 1998 mei-W68 in *Drosophila melanogaster* encodes a Spo11 homolog: evidence that the mechanism for initiating meiotic recombination is conserved. Genes Dev. 12: 2932–2942 9744869 10.1101/gad.12.18.2932 PMC317166

[bib43] McVean G. A. Myers S. R. Hunt S. Deloukas P. Bentley D. R., 2004 The fine-scale structure of recombination rate variation in the human genome. Science 304: 581–584 15105499 10.1126/science.1092500

[bib44] Mehrotra S. McKim K. S., 2006 Temporal analysis of meiotic DNA double-strand break formation and repair in Drosophila females. PLoS Genet. 2: 1883–1897 10.1371/journal.pgen.0020200 PMC1657055 17166055

[bib45] Metzker M. L., 2010 Sequencing technologies—the next generation. Nat. Rev. Genet. 11: 31–46 19997069 10.1038/nrg2626

[bib46] Myers S. Bottolo L. Freeman C. McVean G. Donnelly P., 2005 A fine-scale map of recombination rates and hotspots across the human genome. Science 310: 321–324 16224025 10.1126/science.1117196

[bib47] Myers S. Freeman C. Auton A. Donnelly P. McVean G., 2008 A common sequence motif associated with recombination hot spots and genome instability in humans. Nat. Genet. 40: 1124–1129 19165926 10.1038/ng.213

[bib48] Myers S. Bowden R. Tumian A. Bontrop R. E. Freeman C., 2010 Drive against hotspot motifs in primates implicates the PRDM9 gene in meiotic recombination. Science 327: 876–879 20044541 10.1126/science.1182363 PMC3828505

[bib49] Nielsen R. Paul J. S. Albrechtsen A. Song Y. S., 2011 Genotype and SNP calling from next-generation sequencing data. Nat. Rev. Genet. 12: 443–451 21587300 10.1038/nrg2986 PMC3593722

[bib50] O’Reilly P. F. Birney E. Balding D. J., 2008 Confounding between recombination and selection, and the Ped/Pop method for detecting selection. Genome Res. 18: 1304–1313 18617692 10.1101/gr.067181.107 PMC2493435

[bib51] Portales-Casamar E. Thongjuea S. Kwon A. T. Arenillas D. Zhao X., 2010 JASPAR 2010: the greatly expanded open-access database of transcription factor binding profiles. Nucleic Acids Res. 38: D105–D110 19906716 10.1093/nar/gkp950 PMC2808906

[bib52] Pugh T. J. Delaney A. D. Farnoud N. Flibotte S. Griffith M., 2008 Impact of whole genome amplification on analysis of copy number variants. Nucleic Acids Res. 36: e80 18559357 10.1093/nar/gkn378 PMC2490749

[bib53] Qi J. Wijeratne A. J. Tomsho L. P. Hu Y. Schuster S. C., 2009 Characterization of meiotic crossovers and gene conversion by whole-genome sequencing in *Saccharomyces cerevisiae*. BMC Genomics 10: 475 19832984 10.1186/1471-2164-10-475 PMC2770529

[bib54] Radford S. J. McMahan S. Blanton H. L. Sekelsky J., 2007 a Heteroduplex DNA in meiotic recombination in Drosophila mei-9 mutants. Genetics 176: 63–72 17339219 10.1534/genetics.107.070557 PMC1893050

[bib55] Radford S. J. Sabourin M. M. McMahan S. Sekelsky J., 2007 b Meiotic recombination in Drosophila Msh6 mutants yields discontinuous gene conversion tracts. Genetics 176: 53–62 17339220 10.1534/genetics.107.070367 PMC1893074

[bib56] Schweitzer M. D., 1935 An analytical study of crossing over in *Drosophila melanogaster*. Genetics 20: 497–527 17246774 10.1093/genetics/20.5.497 PMC1208627

[bib57] Singh N. D. Aquadro C. F. Clark A. G., 2009 Estimation of fine-scale recombination intensity variation in the white-echinus interval of *D-melanogaster*. J. Mol. Evol. 69: 42–53 19504037 10.1007/s00239-009-9250-5 PMC2748731

[bib58] Smith P. D. Finnerty V. G. Chovnick A., 1970 Gene conversion in *Drosophila*: non-reciprocal events at the marron-like cistron. Nature 228: 442–444 5482495 10.1038/228442a0

[bib59] Stevison L. S. Noor M. A. F., 2010 Genetic and evolutionary correlates of fine-scale recombination rate variation in *Drosophila persimilis*. J. Mol. Evol. 71: 332–345 20890595 10.1007/s00239-010-9388-1

[bib60] Sun H. Treco D. Schultes N. P. Szostak J. W., 1989 Double-strand breaks at an initiation site for meiotic gene conversion. Nature 338: 87–90 2645528 10.1038/338087a0

[bib61] Szostak J. W. Orr-Weaver T. L. Rothstein R. J. Stahl F. W., 1983 The double-strand-break repair model for recombination. Cell 33: 25–35 6380756 10.1016/0092-8674(83)90331-8

[bib62] Tweedie S. Ashburner M. Falls K. Leyland P. McQuilton P., 2009 FlyBase: enhancing Drosophila Gene Ontology annotations. Nucleic Acids Res. 37: D555–D559 18948289 10.1093/nar/gkn788 PMC2686450

[bib63] Youds J. L. Boulton S. J., 2011 The choice in meiosis—defining the factors that influence crossover or non-crossover formation. J. Cell Sci. 124: 501–513 21282472 10.1242/jcs.074427

