# Supplementary material for: A Whole-Chromosome Analysis of Meiotic Recombination in Drosophila melanogaster
Source: G3 (Bethesda). 2012 Feb 1;2(2):249–60. doi: 10.1534/g3.111.001396 (PMC3284332; doi:10.1534/g3.111.001396)
Supplement: Supporting Information [file supp_2_2_249__index.html]

Supporting Information 

# A Whole-Chromosome Analysis of Meiotic Recombination in *Drosophila melanogaster*

## Supporting Information for Miller *et al.*, 2012

**Files in this Data Supplement:**

- Supporting Information - Figure S1 and Tables S1-S4 (PDF, 257 KB)
- Figure S1 - Cross scheme used to generate males for sequencing and preservation of X chromosomes that have experienced one round of female meiosis (PDF, 58 KB)
- Table S1 - Sequencing Summary (PDF, 70 KB)
- Table S2 - Crossover Primers (PDF, 59 KB)
- Table S3 - Gene Conversion Primers (PDF, 38 KB)
- Table S4 - False Positive Gene Conversion Primers (PDF, 40 KB)
