# Supplementary material for: A Whole-Chromosome Analysis of Meiotic Recombination in Drosophila melanogaster
Source: G3 (Bethesda). 2012 Feb 1;2(2):249–60. doi: 10.1534/g3.111.001396 (PMC3284332; doi:10.1534/g3.111.001396)
Supplement: Supporting Information [file supp_2.2.249_001393SI.pdf]

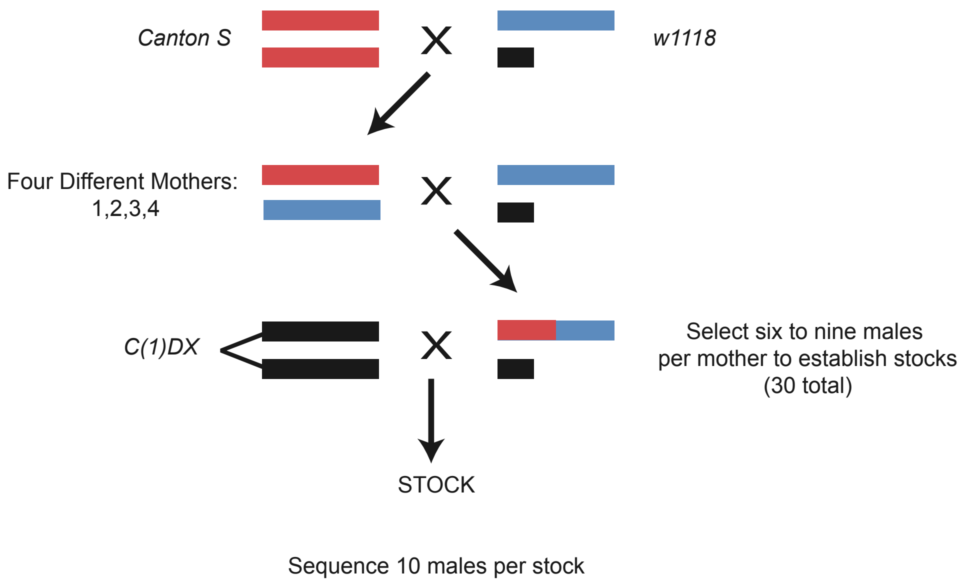

Figure S1 Cross scheme used to generate males for sequencing and preservation of *X* chromosomes that have experienced one round of female meiosis. Isogenized *Canton-S* females and w^{1118} males were crossed and male progeny were then singly mated to *C(1)DX* females for five days before the male was removed and stored at -80°C. Male progeny were collected from each stock as needed for sequencing the recombinant *X* chromosome within the stock.

Table S1 Sequencing Summary

	Lanes	Aligned Reads	Coverage	Average Depth	SNPs	Indels	Total SNPs/Indels	% of Total
**Parental**								
Canton S	2	9,566,323	99%	17.1x	79,045	14,483	93,528	100%
w^{1118}	*2*	10,724,804	99%	19.1x	79,045	14,483	93,528	100%
**Progeny**								
1a	1	2,870,313	95%	5.1x	66,350	2,366	68,716	73%
1b	1	2,352,362	92%	4.2x	63,848	1,532	65,380	70%
1c	1	2,166,375	94%	3.9x	64,695	1,843	66,538	71%
1d	1	2,446,926	92%	4.4x	62,273	1,886	64,159	69%
1e	1	2,703,932	92%	4.8x	62,203	2,047	64,250	69%
1f	1	2,568,717	93%	4.6x	63,715	1,982	65,697	70%
1g	1	1,768,100	83%	3.2x	54,635	879	55,514	59%
2a	1	2,611,195	94%	4.7x	64,456	2,084	66,540	71%
2b	1	2,670,266	94%	4.8x	65,106	2,369	67,475	72%
2c	1	4,429,072	97%	7.9x	68,256	931	69,187	74%
2d	1	1,811,129	85%	3.2x	56,902	1,047	57,949	62%
2e	1	2,382,349	86%	4.2x	57,949	1,337	59,286	63%
2f	1	2,676,214	88%	4.8x	59,175	1,819	60,994	65%
2g	1	1,749,264	80%	3.1x	52,186	1,013	53,199	57%
2h	1	2,610,803	90%	4.7x	60,474	1,682	62,156	66%
3a	1	2,958,632	96%	5.3x	67,572	2,530	70,102	75%
3b	1	2,729,282	94%	4.9x	64,309	1,820	66,129	71%
3c	1	5,980,205	99%	10.7x	71,079	2,563	73,642	79%
3d	1	4,054,558	97%	7.2x	68,312	2,494	70,806	76%
3e	1	4,073,933	98%	7.3x	66,681	2,470	69,151	74%
3f	1	4,224,625	98%	7.5x	69,354	2,470	71,824	77%
3g	1	2,642,452	94%	4.7x	64,569	2,013	66,582	71%
3h	1	3,086,857	97%	5.5x	66,156	3,050	69,206	74%
3i	1	4,978,091	98%	8.9x	70,196	3,632	73,828	79%
4a	1	4,951,138	98%	8.8x	70,161	4,497	74,658	80%
4b	1	3,639,466	96%	6.5x	66,881	2,945	69,826	75%
4c	1	2,236,037	90%	4.0x	64,688	1,104	65,792	70%

4d	1	3,158,135	94%	5.6x	64,967	2,362	67,329	72%
4e	1	4,542,615	97%	8.1x	68,180	3,695	71,875	77%
4f	1	3,751,727	96%	6.7x	66,799	3,138	69,937	75%

Average depth is defined as number of aligned reads multiplied by the read length divided by the size of the X chromosome. Percent coverage is the amount of the X chromosome covered with at least 1 read. SNPs and indels for parental lines are defined as SNPs and indels compared to the published reference that differ between the two lines with a quality score >= 30. SNPs and indels for progeny lines are defined as SNPs and indels that exist in the parents that were also seen in the progeny with a quality score >= 30.

Table S2   Crossover Primers

Progeny	Base	Left Primer	Base	Right Primer
1a	6,601,201	caccttccaccttccacct	6,602,014	atgcatgccaagatgtgaac
1b	8,000,672	gtgcattcgtgtgcattctt	8,001,371	ctgtttgatcggctcttttt
1c	12,813,050	ccatcaaagcatcaacacca	12,813,882	ttacgtgggcatgactagga
	12,813,930	ttttcgctcgcaacaataga	12,814,137	ttacgtgggcatgactagga
	12,813,399	ttaaagcaaggacccagcaa	12,814,631	ttgcactaacccaattacatcg
1d	15,696,200	cccaagtcagcccatcttta	15,698,261	agctctttggttggcttgaa
1e	5,441,030	gggcttgtcacacacacatc	5,441,986	tgtcagctccttccttcgat
1e	16,511,710	gcggcttcagtgagtcaagt	16,514,291	cggcatggtgtattatgcaa
	16,512,296	tatgcaactggcaaaaggtg	16,512,489	cgtgatcgatccattcaaaa
	16,513,120	ttcctttcgtttcgcactct	16,513,322	caaagcaacttccgcttctt
	16,513,698	cttattacgggccaaacgtg	16,513,860	aacaactcaccaggccaaac
2a	11,992,619	gaatggcatggaatggaatc	11,996,385	tggcatatccactgttttgc
	11,993,091	ccaaggatctggcagaaaat	11,993,325	tgcgacttcaatggatcaaa
	11,993,886	ctgcactgggaaaaacgagt	11,994,084	ccacgaatggcgaagtaaat
	11,994,587	tggtgaacagtgttgggaaa	11,994,818	cagcacaacgtcaaaaagga
	11,995,628	aatcctgcatccacctcatc	11,995,856	gtatcgccaaggagtgggta
2a	19,451,000	gtgtgtcgacctggtccttt	19,453,357	aagttttaaataaagtttgcggtttt
	19,451,532	cgggattgtggaaagtgtct	19,451,728	gatccccacggtatcttcaa
	19,452,199	gaagaaggacgaacggatga	19,452,428	gccgacaagtttgttgtcac
	19,453,041	ggaatcccaacacaacgaat	19,453,244	tctgatagggaaggcactcg
2c	8,833,961	tgtggcagcgtttattgttt	8,834,509	gcccctcatcacgaaactg
2d	2,413,018	tattttgtctgccccctttg	2,414,432	gggagctgcactgtgttgt
	2,413,649	ctttcgaaaccatcccaaaa	2,413,798	ttggtggatttcaatgcaga
3a	4,860,618	gggattgttctcaggctcaa	4,861,822	tgagatgtttagcagcagtga
3b	8,862,505	tgaaatcaaggcgaatagca	8,863,092	tttcttctcggctgctttgt
	8,862,621	ttcgcttatttccgctcatt	8,863,678	aagcttttgggcatgatttg
	8,863,918	tgattgtctcgagcagttgg	8,864,477	aaacaccaagtgtgcagcaa
4a	11,968,316	gatgatgagcagcagcaaga	11,970,690	attctgccaccctgtctgtc
	11,969,629	gcacagacagcgagactgag	11,969,821	ttgactggcttgcaaatacg
	11,970,201	cccccatttgtatgtgtgtg	11,970,398	ctcaatcttcggctcgaaac
4a	19,291,414	ggttgcttaagttgcttagatgg	19,293,324	aaatgatagcggagagaaccaa
	19,291,958	cagaggctaacccgttgaag	19,292,145	ttgaagtccgttctggttcc
	19,292,361	cccgtccaatttgaaacaat	19,292,610	cgggctaagccagactacag
	19,292,784	atcgttggctattgcacgtc	19,292,977	ggtggcgaacatttgtatcc

Primers used to validate the crossovers observed in the WGS data. Multiple primers were used to sequence segments which spanned more than approximately 500bp. One PCR reaction was done for each segment. Validations were repeated at least in triplicate.

Table S3 Gene Conversion Primers

Progeny	Base	Left Primer	Base	Right Primer
3c	9,157,347	tgcacataaatgtggttcacaa	9,161,322	ccctcaacggacgaagtaat
	9,158,050	gtcgcagcagttcttcatca	9,158,290	ccaaggcactagcactgtca
	9,159,091	gatgccataggcacccttac	9,159,241	tggaaagcattttcgatgtg
	9,160,019	cgggagcacaaataaaaagc	9,160,268	agcatccaagagctttacgc
	9,160,729	atggactccgtttgaaatgc	9,160,955	ctggtaagtgacgtgctcca
3c	19,520,701	tctctccagccgaggtttg	19,521,688	cgcctaagttgaatagcctagc
3d	10,913,917	ccttcatccaaatccgactg	10,916,491	gatagcccccaaaaagggta
	10,914,843	aagagccgtcaacaccttgt	10,915,092	actggcgatgagaggaagaa
	10,915,861	acagtgcagggaaaaccaac	10,916,020	tgccattgattgaattgtacg
2b	15,151,200	gctgctcatcaccagtcaac	15,152,200	cccgtgtggaaatgaaactc
1e	6,633,000	cagatcggtgcgtgatgtc	6,633,750	tcgaaccactaaagcacctg

Primers used to validate the real gene conversions observed in the WGS data. Multiple primers were used to sequence segments which spanned more than approximately 500bp. One PCR reaction was done for each segment. Validations were repeated at least in triplicate.

Table S4   False Positive Gene Conversion Primers

Progeny	SNP	Left Primer	Right Primer
3b	164,089	ttggatccactttcaggagaa	ctaagagcgcgaatcaaacc
2b	237,888	cgagaagccgtaaaaagcac	tgcactgggacgcactaata
1b	268,501	ttgcgataaaagcacactgc	gcctgtcatgaacgcagtc
2e	268,501	cgacgcaaagggaagataag	ctaccatcgaggggatgaaa
1f	1,200,997	gcatttcgtggagaaagctc	cgggtactcgacgtattttga
3e	3,762,602	ttttgcactgatttgcgttg	ggggtgtccactaattcagc
1e	5,189,307	tcatattcagcactcaaagtgga	caattggcgtaagggtattca
3e	6,876,237	agcgaaacagctggctatga	tggcaatcaaagagaatctgg
3e	7,647,353	acgttgcggtcagtttaagg	atccattggccgactttagc
2h	8,250,117	caatgtttgctgcccaatta	aaaaagccactcacgaggaa
3g	8,250,117	caataattggaattagaatga	tccctaaaaagccactcacg
3h	8,804,104	tgagcactcgaaataatgaaatg	cagaaaacaattggccaaaaa
3g	8,804,104	tgagcactcgaaataatgaaatg	cagaaaacaattggccaaaaa
3b	8,874,618	gccaaaaattcgacacttacg	tggaaataaatactaaatggcct
3f	8,911,293	ctgagctttaaataagctttacata	acccaaatagcttgctttgc
4b	8,911,293	ctgagctttaaataagctttacata	acccaaatagcttgctttgc
1c	9,215,074	cgcaacgtgaccacatattc	gtggtgagtccctccatacg
3g	12,247,068	cgggcaatgtcaacgtctat	tgcacacagttacgaccact
2b	12,707,830	tttttggaacataccattcacaa	tctgtgtgtgagtggcgagt
1e	17,689,327	gggacacgattttatcagca	ctgctcgatgttgcgtttta
1d	18,999,675	gcactgcgggaaacaaatag	agcaatgagtgtcttggcttc
2g	19,306,566	aagctaatagatgtgctgtgcaa	aaggcaacttagccctggtt
2d	20,586,835	cctgcagccttttgaaggta	tcgtttttgctttcagcactt

Primers used to check candidate gene conversions which turned out to be false positives based on the WGS data.

One PCR reaction was done for each segment. Validations were repeated at least in triplicate.
