# Supplementary material for: A Whole-Chromosome Analysis of Meiotic Recombination in Drosophila melanogaster
Source: G3 (Bethesda). 2012 Feb 1;2(2):249–60. doi: 10.1534/g3.111.001396 (PMC3284332; doi:10.1534/g3.111.001396)
Supplement: Supporting Information [file supp_2.2.249_FigureS1.pdf]

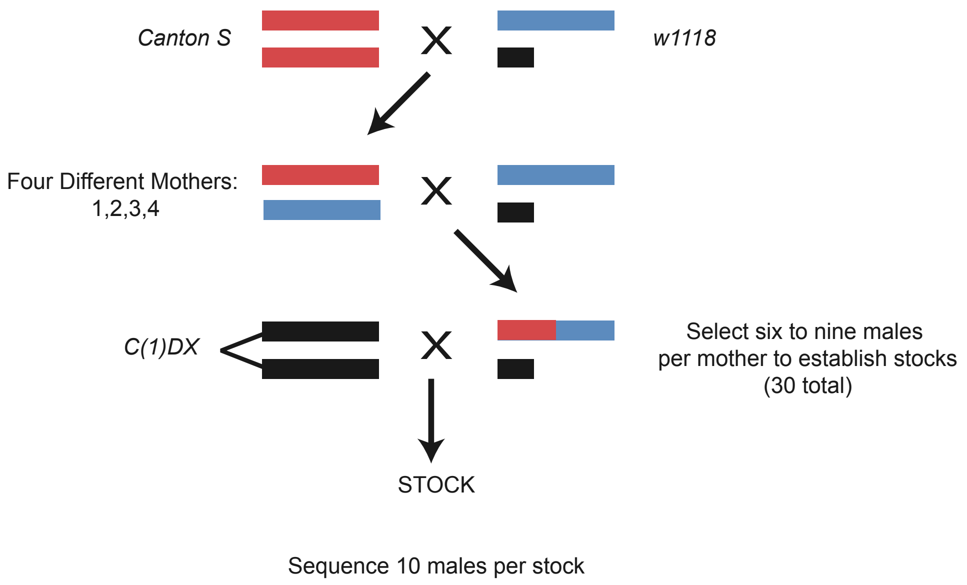

Figure S1 Cross scheme used to generate males for sequencing and preservation of *X* chromosomes that have experienced one round of female meiosis. Isogenized *Canton-S* females and w^{1118} males were crossed and male progeny were then singly mated to *C(1)DX* females for five days before the male was removed and stored at -80°C. Male progeny were collected from each stock as needed for sequencing the recombinant *X* chromosome within the stock.
