# Supplementary material for: A Whole-Chromosome Analysis of Meiotic Recombination in Drosophila melanogaster
Source: G3 (Bethesda). 2012 Feb 1;2(2):249–60. doi: 10.1534/g3.111.001396 (PMC3284332; doi:10.1534/g3.111.001396)
Supplement: Supporting Information [file supp_2.2.249_TableS1.pdf]

Table S1 Sequencing Summary

	Lanes	Aligned Reads	Coverage	Average Depth	SNPs	Indels	Total SNPs/Indels	% of Total
**Parental**								
Canton S	2	9,566,323	99%	17.1x	79,045	14,483	93,528	100%
w[1118]	*2*	10,724,804	99%	19.1x	79,045	14,483	93,528	100%
**Progeny**								
1a	1	2,870,313	95%	5.1x	66,350	2,366	68,716	73%
1b	1	2,352,362	92%	4.2x	63,848	1,532	65,380	70%
1c	1	2,166,375	94%	3.9x	64,695	1,843	66,538	71%
1d	1	2,446,926	92%	4.4x	62,273	1,886	64,159	69%
1e	1	2,703,932	92%	4.8x	62,203	2,047	64,250	69%
1f	1	2,568,717	93%	4.6x	63,715	1,982	65,697	70%
1g	1	1,768,100	83%	3.2x	54,635	879	55,514	59%
2a	1	2,611,195	94%	4.7x	64,456	2,084	66,540	71%
2b	1	2,670,266	94%	4.8x	65,106	2,369	67,475	72%
2c	1	4,429,072	97%	7.9x	68,256	931	69,187	74%
2d	1	1,811,129	85%	3.2x	56,902	1,047	57,949	62%
2e	1	2,382,349	86%	4.2x	57,949	1,337	59,286	63%
2f	1	2,676,214	88%	4.8x	59,175	1,819	60,994	65%
2g	1	1,749,264	80%	3.1x	52,186	1,013	53,199	57%
2h	1	2,610,803	90%	4.7x	60,474	1,682	62,156	66%
3a	1	2,958,632	96%	5.3x	67,572	2,530	70,102	75%
3b	1	2,729,282	94%	4.9x	64,309	1,820	66,129	71%
3c	1	5,980,205	99%	10.7x	71,079	2,563	73,642	79%
3d	1	4,054,558	97%	7.2x	68,312	2,494	70,806	76%
3e	1	4,073,933	98%	7.3x	66,681	2,470	69,151	74%
3f	1	4,224,625	98%	7.5x	69,354	2,470	71,824	77%
3g	1	2,642,452	94%	4.7x	64,569	2,013	66,582	71%
3h	1	3,086,857	97%	5.5x	66,156	3,050	69,206	74%
3i	1	4,978,091	98%	8.9x	70,196	3,632	73,828	79%
4a	1	4,951,138	98%	8.8x	70,161	4,497	74,658	80%
4b	1	3,639,466	96%	6.5x	66,881	2,945	69,826	75%
4c	1	2,236,037	90%	4.0x	64,688	1,104	65,792	70%

4d	1	3,158,135	94%	5.6x	64,967	2,362	67,329	72%
4e	1	4,542,615	97%	8.1x	68,180	3,695	71,875	77%
4f	1	3,751,727	96%	6.7x	66,799	3,138	69,937	75%

Average depth is defined as number of aligned reads multiplied by the read length divided by the size of the X chromosome. Percent coverage is the amount of the X chromosome covered with at least 1 read. SNPs and indels for parental lines are defined as SNPs and indels compared to the published reference that differ between the two lines with a quality score >= 30. SNPs and indels for progeny lines are defined as SNPs and indels that exist in the parents that were also seen in the progeny with a quality score >= 30.
