# Supplementary material for: A Whole-Chromosome Analysis of Meiotic Recombination in Drosophila melanogaster
Source: G3 (Bethesda). 2012 Feb 1;2(2):249–60. doi: 10.1534/g3.111.001396 (PMC3284332; doi:10.1534/g3.111.001396)
Supplement: Supporting Information [file supp_2.2.249_TableS2.pdf]

Table S2 Crossover Primers

Progeny	Base	Left Primer	Base	Right Primer
1a	6,601,201	caccttccaccttccacct	6,602,014	atgcatgccaagatgtgaac
1b	8,000,672	gtgcattcgtgtgcattctt	8,001,371	ctgtttgatcggctcttttt
1c	12,813,050	ccatcaaagcatcaacacca	12,813,882	ttacgtgggcatgactagga
	12,813,930	ttttcgctcgcaacaataga	12,814,137	ttacgtgggcatgactagga
	12,813,399	ttaaagcaaggacccagcaa	12,814,631	ttgcactaacccaattacatcg
1d	15,696,200	cccaagtcagcccatcttta	15,698,261	agctctttggttggcttgaa
1e	5,441,030	gggcttgtcacacacacatc	5,441,986	tgtcagctccttccttcgat
1e	16,511,710	gcggcttcagtgagtcaagt	16,514,291	cggcatggtgtattatgcaa
	16,512,296	tatgcaactggcaaaaggtg	16,512,489	cgtgatcgatccattcaaaa
	16,513,120	ttcctttcgtttcgcactct	16,513,322	caaagcaacttccgcttctt
	16,513,698	cttattacgggccaaacgtg	16,513,860	aacaactcaccaggccaaac
2a	11,992,619	gaatggcatggaatggaatc	11,996,385	tggcatatccactgttttgc
	11,993,091	ccaaggatctggcagaaaat	11,993,325	tgcgacttcaatggatcaaa
	11,993,886	ctgcactgggaaaaacgagt	11,994,084	ccacgaatggcgaagtaaat
	11,994,587	tggtgaacagtgttgggaaa	11,994,818	cagcacaacgtcaaaaagga
	11,995,628	aatcctgcatccacctcatc	11,995,856	gtatcgccaaggagtgggta
2a	19,451,000	gtgtgtcgacctggtccttt	19,453,357	aagttttaaataaagtttgcggtttt
	19,451,532	cgggattgtggaaagtgtct	19,451,728	gatccccacggtatcttcaa
	19,452,199	gaagaaggacgaacggatga	19,452,428	gccgacaagtttgttgtcac
	19,453,041	ggaatcccaacacaacgaat	19,453,244	tctgatagggaaggcactcg
2c	8,833,961	tgtggcagcgtttattgttt	8,834,509	gcccctcatcacgaaactg
2d	2,413,018	tattttgtctgccccctttg	2,414,432	gggagctgcactgtgttgt
	2,413,649	ctttcgaaaccatcccaaaa	2,413,798	ttggtggatttcaatgcaga
3a	4,860,618	gggattgttctcaggctcaa	4,861,822	tgagatgtttagcagcagtga
3b	8,862,505	tgaaatcaaggcgaatagca	8,863,092	tttcttctcggctgctttgt
	8,862,621	ttcgcttatttccgctcatt	8,863,678	aagcttttgggcatgatttg
	8,863,918	tgattgtctcgagcagttgg	8,864,477	aaacaccaagtgtgcagcaa
4a	11,968,316	gatgatgagcagcagcaaga	11,970,690	attctgccaccctgtctgtc
	11,969,629	gcacagacagcgagactgag	11,969,821	ttgactggcttgcaaatacg
	11,970,201	cccccatttgtatgtgtgtg	11,970,398	ctcaatcttcggctcgaaac
4a	19,291,414	ggttgcttaagttgcttagatgg	19,293,324	aaatgatagcggagagaaccaa
	19,291,958	cagaggctaacccgttgaag	19,292,145	ttgaagtccgttctggttcc
	19,292,361	cccgtccaatttgaaacaat	19,292,610	cgggctaagccagactacag
	19,292,784	atcgttggctattgcacgtc	19,292,977	ggtggcgaacatttgtatcc

Primers used to validate the crossovers observed in the WGS data. Multiple primers were used to sequence segments which spanned more than approximately 500bp. One PCR reaction was done for each segment. Validations were repeated at least in triplicate.
