# Supplementary material for: A Whole-Chromosome Analysis of Meiotic Recombination in Drosophila melanogaster
Source: G3 (Bethesda). 2012 Feb 1;2(2):249–60. doi: 10.1534/g3.111.001396 (PMC3284332; doi:10.1534/g3.111.001396)
Supplement: Supporting Information [file supp_2.2.249_TableS3.pdf]

Table S3 Gene Conversion Primers

Progeny	Base	Left Primer	Base	Right Primer
3c	9,157,347	tgcacataaatgtggttcacaa	9,161,322	ccctcaacggacgaagtaat
	9,158,050	gtcgcagcagttcttcatca	9,158,290	ccaaggcactagcactgtca
	9,159,091	gatgccataggcacccttac	9,159,241	tggaaagcattttcgatgtg
	9,160,019	cgggagcacaaataaaaagc	9,160,268	agcatccaagagctttacgc
	9,160,729	atggactccgtttgaaatgc	9,160,955	ctggtaagtgacgtgctcca
3c	19,520,701	tctctccagccgaggtttg	19,521,688	cgcctaagttgaatagcctagc
3d	10,913,917	ccttcatccaaatccgactg	10,916,491	gatagcccccaaaaagggta
	10,914,843	aagagccgtcaacaccttgt	10,915,092	actggcgatgagaggaagaa
	10,915,861	acagtgcagggaaaaccaac	10,916,020	tgccattgattgaattgtacg
2b	15,151,200	gctgctcatcaccagtcaac	15,152,200	cccgtgtggaaatgaaactc
1e	6,633,000	cagatcggtgcgtgatgtc	6,633,750	tcgaaccactaaagcacctg

Primers used to validate the real gene conversions observed in the WGS data. Multiple primers were used to sequence segments which spanned more than approximately 500bp. One PCR reaction was done for each segment. Validations were repeated at least in triplicate.
