# Supplementary material for: A Whole-Chromosome Analysis of Meiotic Recombination in Drosophila melanogaster
Source: G3 (Bethesda). 2012 Feb 1;2(2):249–60. doi: 10.1534/g3.111.001396 (PMC3284332; doi:10.1534/g3.111.001396)
Supplement: Supporting Information [file supp_2.2.249_TableS4.pdf]

Table S4 False Positive Gene Conversion Primers

Progeny	SNP	Left Primer	Right Primer
3b	164,089	ttggatccactttcaggagaa	ctaagagcgcgaatcaaacc
2b	237,888	cgagaagccgtaaaaagcac	tgcactgggacgcactaata
1b	268,501	ttgcgataaaagcacactgc	gcctgtcatgaacgcagtc
2e	268,501	cgacgcaaagggaagataag	ctaccatcgaggggatgaaa
1f	1,200,997	gcatttcgtggagaaagctc	cgggtactcgacgtattttga
3e	3,762,602	ttttgcactgatttgcgttg	ggggtgtccactaattcagc
1e	5,189,307	tcatattcagcactcaaagtgga	caattggcgtaagggtattca
3e	6,876,237	agcgaaacagctggctatga	tggcaatcaaagagaatctgg
3e	7,647,353	acgttgcggtcagtttaagg	atccattggccgactttagc
2h	8,250,117	caatgtttgctgcccaatta	aaaaagccactcacgaggaa
3g	8,250,117	caataattggaattagaatga	tccctaaaaagccactcacg
3h	8,804,104	tgagcactcgaaataatgaaatg	cagaaaacaattggccaaaaa
3g	8,804,104	tgagcactcgaaataatgaaatg	cagaaaacaattggccaaaaa
3b	8,874,618	gccaaaaattcgacacttacg	tggaaataaatactaaatggcct
3f	8,911,293	ctgagctttaaataagctttacata	acccaaatagcttgctttgc
4b	8,911,293	ctgagctttaaataagctttacata	acccaaatagcttgctttgc
1c	9,215,074	cgcaacgtgaccacatattc	gtggtgagtccctccatacg
3g	12,247,068	cgggcaatgtcaacgtctat	tgcacacagttacgaccact
2b	12,707,830	tttttggaacataccattcacaa	tctgtgtgtgagtggcgagt
1e	17,689,327	gggacacgattttatcagca	ctgctcgatgttgcgtttta
1d	18,999,675	gcactgcgggaaacaaatag	agcaatgagtgtcttggcttc
2g	19,306,566	aagctaatagatgtgctgtgcaa	aaggcaacttagccctggtt
2d	20,586,835	cctgcagccttttgaaggta	tcgtttttgctttcagcactt

Primers used to check candidate gene conversions which turned out to be false positives based on the WGS data.

One PCR reaction was done for each segment. Validations were repeated at least in triplicate.
